# Dopamine-Conjugated Methacrylated Gelatin Hydrogel—Physical, Mechanical, and Biological Properties

**DOI:** 10.3390/gels11070499

**Published:** 2025-06-26

**Authors:** Weiwen Lu, Maedeh Rahimnejad, Beatriz Ometto Sahadi, Marco C. Bottino

**Affiliations:** 1 Department of Biomedical Engineering, College of Engineering, University of Michigan, Ann Arbor, MI 48109, USA; victorlu@umich.edu; 2 Department of Cariology, Restorative Sciences, and Endodontics, School of Dentistry, University of Michigan, 1011 N. University (Room 2303), Ann Arbor, MI 48109, USA; rmaedeh@umich.edu (M.R.); bia_sahadi@live.com (B.O.S.); 3 Department of Restorative Dentistry, Piracicaba Dental School, University of Campinas, Piracicaba 13414-903, SP, Brazil

**Keywords:** wound closure, tissue adhesive, methacrylated gelatin (GelMA), dopamine (DOPA)

## Abstract

This study develops and characterizes GelMA–dopamine conjugates as novel tissue adhesives, offering an alternative to sutures. GelMA was synthesized at 5%, 10%, and 15% (*w*/*v*) with medium and high dopamine (DOPA) conjugation. Adhesives were evaluated for swelling, degradation, mechanical strength, and cytocompatibility using AlamarBlue assays and F-actin staining to assess cell viability and adhesion. Our findings indicate that DOPA conjugation significantly reduced the swelling ratio while increasing the biodegradation rate, resulting in enhanced release of free methacrylate groups over time. The mechanical properties and adhesion capabilities showed a complex relationship with DOPA substitution. Notably, the formulation containing 10% GelMA with high dopamine conjugation (HD) exhibited superior adhesion and mechanical strength. All formulations demonstrated shear-thinning behavior and recovery, making them suitable for injection and bioprinting applications. Although increased DOPA levels negatively affected crosslinking, the optimal formulation achieved a balance between adhesion and gel concentration. Rapid crosslinking was achieved within five minutes, enhancing the material’s suitability for clinical applications. In vitro cell-based assays confirmed the non-cytotoxic nature of the optimal adhesives, with metabolic activity showing significant increases over a 7-day period. These advancements support the development of improved tissue adhesives, potentially reducing reliance on sutures and enhancing wound healing outcomes.

## 1. Introduction

Medical suturing is a critical procedure for wound closure in a variety of healthcare settings, yet it presents several inherent challenges [1]. The process is often time-consuming and expensive and demands advanced technical skills, typically requiring specialized medical personnel [1]. These limitations have spurred increasing interest in bioadhesives as a viable alternative for wound closure. Bioadhesives offer a simpler and more cost-effective solution, particularly for less complex wounds [2]. An ideal bioadhesive would possess strong skin-binding properties, promote cellular proliferation, and biodegrade in alignment with the body’s natural tissue regeneration processes [3]. The development of such materials holds significant promise for alleviating healthcare burdens and enhancing patient comfort.

Numerous innovative efforts have been made to develop bioadhesives that facilitate wound closure. For instance, Jeon et al. introduced the light-activated, mussel protein-based bioadhesive (LAMBA), a bioadhesive derived from mussel proteins, demonstrating exceptional wet tissue adhesion properties [4]. Similarly, Zhu and colleagues created a glue composed of bovine serum albumin, citric acid, and DOPA, achieving adhesion strength ten times greater than commercially available fibrin glue bioadhesives [5]. Additionally, Shagan et al. developed a bioadhesive system utilizing four-armed N-hydroxysuccinimide-modified polycaprolactone (star-PCL-NHS), which is stored in a melt gun and rapidly solidifies upon application to the wound surface [3]. Researchers demonstrated a direct correlation between adhesive strength and DOPA content, peaking moderately before declining [6]. While these formulations exhibited promising adhesive properties compared to fibrin glue, the long curing time associated with the crosslinking process remains a limitation [6]. Highlighting the potential of bioadhesives, it has been observed that they can significantly modulate the wound closure process, leading to notable biological outcomes. However, none of the currently commercialized adhesives fully meet performance expectations [7]. Developing an adhesive system that functions effectively on wet surfaces while enhancing tissue repair remains challenging [7,8].

**Figure 1:**
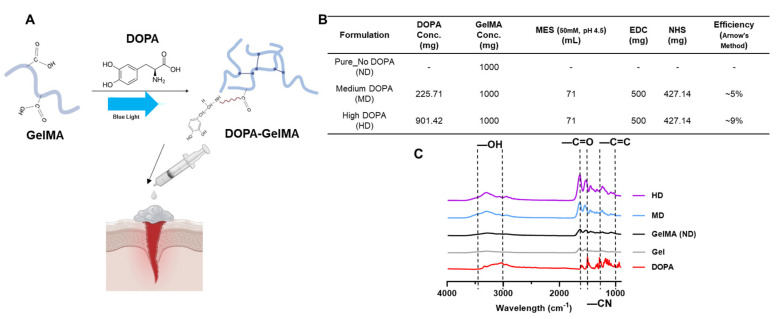
(**A**) Schematic representation illustrating the incorporation of DOPA into the carboxyl groups of GelMA, enhancing adhesive characteristics; (**B**) details of materials used to synthesize different groups used for the experiment and efficiency of DOPA functional group conjugation using Arnow’s method; (**C**) FTIR spectra comparing GelMA hydrogels with medium (MD) and high (HD) concentrations of DOPA to GelMA, gelatin (Gel), and DOPA, highlighting the presence of DOPA-specific peaks.

Building on these advancements, gelatin methacryloyl (GelMA) has emerged as a promising biomaterial, which is known for its biocompatibility and customizable mechanical properties. In this study, we developed a GelMA-based bioadhesive modified with dopamine (DOPA) to enhance adhesive strength (Figure 1A). We comprehensively assessed the bioadhesive’s mechanical performance through shear thinning, amplitude sweep, temperature sweep, recovery, tensile, and compression tests. Additionally, we validated its biocompatibility by evaluating cell viability using gingival stem cells, emphasizing the material’s potential to support cellular activity and promote tissue regeneration. This research represents a significant advancement in developing effective, degradable bioadhesives, potentially streamlining wound closure procedures in clinical settings.

## 2. Results and Discussion

### 2.1. Chemical Characterizations

The FTIR spectra of GelMA and DOPA-conjugated GelMA (DOPA-GelMA) are presented in Figure 1C, where distinct peaks reflect the functional groups within the materials and confirm the successful conjugation of DOPA to GelMA. In the GelMA spectrum, characteristic peaks align with those expected for gelatin methacryloyl: (1) The amide I band at 1650–1680 cm^−1^ is attributed to C=O stretching, a hallmark of gelatin-based materials. (2) The amide II band near 1550 cm^−1^ corresponds to N-H bending, indicating the presence of peptide linkages in the gelatin backbone. (3) A peak around 1630–1640 cm^−1^, associated with methacrylate C=C stretching [9], verifies the successful methacrylation of gelatin. (4) The amide III band at 1200–1300 cm^−1^, indicative of C-N stretching and N-H bending, further confirms the integrity of GelMA’s protein structure.

With the conjugation of DOPA, DOPA-GelMA shows additional or shifted peaks compared to GelMA alone, evidencing the presence of catechol groups: (1) A broadened hydroxyl (–OH) stretching peak is observed around 3200–3400 cm^−1^, which is characteristic of the phenolic –OH groups introduced by DOPA. This feature indicates enhanced hydrophilicity, which is a beneficial property for cell interaction and bioactivity in tissue engineering applications [10]. (2) A new peak in the 1260–1300 cm^−1^ range corresponds to C-O stretching vibrations, confirming the integration of DOPA’s hydroxyl functionalities within the GelMA matrix. (3) Notably, an increase in the intensity of aromatic C=C stretching vibrations is seen at approximately 1450–1600 cm^−1^, signaling the successful incorporation of the catechol rings from DOPA into the GelMA backbone [8].

**Figure 2:**
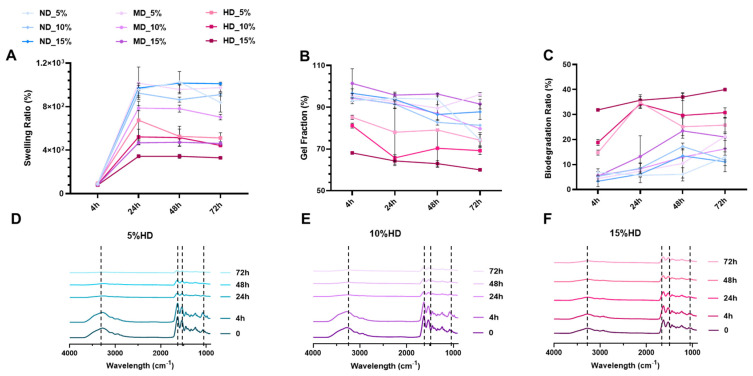
(**A**–**C**) Swelling capacity, gel fraction, and degradation profile of GelMA hydrogels (5%, 10%, and 15% *w*/*v*) without (ND) and with medium (MD) and high DOPA (HD)-concentrated conjugation in non-collagenous solution (PBS buffer) over 3 days at 37 °C (n = 6, mean ± SD) and (**D**–**F**) FTIR spectra of HD hydrogels (GelMA 5%, 10%, and 15% *w*/*v*) during 3 days of degradation.

The observed shifts and additional peaks in DOPA-GelMA compared to GelMA demonstrate that DOPA conjugation successfully modified the polymer. These changes are expected to influence the mechanical, adhesive, and biological properties of GelMA, thereby enhancing its applicability for tissue engineering [11]. Specifically, the catechol groups from DOPA are expected to enhance GelMA’s cell-adhesion properties and mechanical robustness, as they are known to promote interaction with cellular receptors and strengthen the hydrogel matrix. Moreover, introducing hydrophilic –OH groups suggests that DOPA-GelMA hydrogels may exhibit increased swelling behavior and cell encapsulation efficiency relative to non-modified GelMA [12,13]. However, the incorporation of DOPA functional groups in GelMA resulted in a decreased swelling ratio (Figure 2A). The swelling ratio was reduced as we introduced more DOPA: the DOPA forces the GelMA chains to align in a particular way. The OH group, although hydrophilic, paradoxically does not increase the swelling ratio because H_2_O is blocked by the “shell” created by the GelMA chain. This decrease also suggests that DOPA conjugation enhances the density of the crosslinked network within the hydrogel [14]. The catechol groups in DOPA are known for their ability to participate in additional physical and chemical crosslinking interactions, which likely reduce the free volume available for water absorption within the matrix. The tighter network structure restricts the hydrogel’s expansion in an aqueous environment [15,16], resulting in lower swelling ratios than unmodified GelMA. This reduced swelling behavior may benefit applications requiring structural stability and controlled release properties.

### 2.2. Physiochemical and Mechanical Properties

All formulations were monitored for swelling, gel fraction, and mass loss as presented in Figure 2A–C. The data shown in Figure 2B indicate a reduction in the gel fraction following DOPA conjugation. The gel fraction represents the portion of the hydrogel that remains insoluble after crosslinking, and a decrease suggests a lower overall crosslinking efficiency in DOPA-GelMA. This reduction may result from the catechol groups in DOPA potentially competing with methacrylate groups during crosslinking [17]. DOPA’s presence could lead to non-covalent interactions that lower the effective crosslinking density achieved by visible light, UV, or chemical crosslinking, thus reducing the gel fraction [18].

Notably, DOPA also features an aromatic ring structure, which is hydrophobic. When we consider how densely packed the system would be if we were trying to entangle multiple GelMA chains together to form a 3D structure in space, intuitively, the large aromatic rings would be forced farther away from the center of interactions due to the steric effect to release spatial strains [19,20]. These steric effects reduce the accessibility of functional groups on the GelMA (e.g., methacryloyl groups or other reactive sites) for effective crosslinking, particularly during photopolymerization. The spatial hindrance becomes more pronounced as the degree of DOPA substitution increases, further limiting the network formation. DOPA contains catechol groups capable of hydrogen bonding and π-π interactions [21]. While these interactions can contribute to secondary stabilization, they can also compete with methacryloyl crosslinking. The steric arrangement of DOPA moieties may prevent these secondary interactions from aligning effectively, leading to a less compact network [22,23].

Whereas the solubility of free DOPA is known to be moderate in aqueous environments due to its zwitterionic nature, its behavior can change significantly upon conjugation with polymers. Several studies have demonstrated that DOPA incorporation can influence the overall solubility and hydrophilic–hydrophobic balance of polymer networks, depending on the chemistry and degree of modification [24]. For example, DOPA-modified polymers have shown reduced solubility in some systems due to increased hydrophobic interactions and intermolecular crosslinking via catechol groups [25]. In our hydrogel system, the observed changes in swelling behavior and degradation may be partially attributed to such effects, suggesting that DOPA not only contributes to adhesion but also alters network structure and water interactions. These findings are consistent with previous reports on DOPA–polymer conjugates and support our interpretation of the hydrogel’s physicochemical behavior.

The increased biodegradation rate observed in DOPA-GelMA can be attributed to the crosslinking characteristics imparted by DOPA (Figure 2C). A lower crosslinking density tends to make the hydrogel matrix more susceptible to enzymatic degradation [26]. Additionally, the catechol groups of DOPA may act as steric hindrances, increasing the hydrogel’s susceptibility to hydrolytic or enzymatic degradation within a biological environment. This property may be beneficial for applications where controlled degradation is needed, such as in temporary scaffolds for tissue engineering, as it allows for gradual matrix resorption and replacement by new tissue [27]. We note that the 72 h degradation study (Figure 2C) was designed as an accelerated in vitro degradation test in PBS (a simple buffer lacking enzymes). By 72 h, the DOPA-rich hydrogels exhibited accelerated degradation, characterized by a higher mass loss percentage compared to the DOPA-free gels (control); however, they were not fully dissolved.

These observations collectively highlight the impact of DOPA conjugation on the crosslinking dynamics of GelMA. The decreasing profile in swelling ratio and gel fraction, coupled with the increased biodegradation rate, highlights a more complex crosslinking network within DOPA-GelMA that balances covalent and physical crosslinks. While the catechol groups of DOPA contribute additional physical crosslinks, they may simultaneously hinder some methacrylate-based covalent crosslinking. As a result, DOPA-GelMA hydrogels present unique properties that could make them well-suited for applications requiring moderate stability with gradual degradation, providing a tailored approach for tissue engineering where scaffold remodeling is critical.

Figure 2D–F present FTIR spectra tracking the biodegradation of hydrogels with high DOPA (HD) conjugation across varying concentrations. We chose HD for the spectral analysis because high DOPA samples showed the most pronounced changes in characteristic peaks during degradation. These results reveal significant degradation within the methacrylate groups as biodegradation progresses, indicating preferential breakdown within this region of the polymer structure. The characteristic peak of methacrylate C=C stretching around 1630–1640 cm^−1^ exhibits a marked decrease in intensity over time, especially in hydrogels with higher DOPA concentrations. This decline suggests that the methacrylate regions, critical to the crosslinked network, are more susceptible to degradation pathways through hydrolytic or enzymatic mechanisms. The reduced stability of methacrylated groups could be attributed to the high DOPA conjugation, which may interfere with or weaken methacrylate crosslinks during the initial formation, resulting in a more easily degradable structure [12,26]. Additionally, the presence of DOPA catechol groups could influence the degradation profile by enhancing hydrogel hydrophilicity. Higher hydrophilicity accelerates water uptake, which may facilitate the breakdown of ester bonds in methacrylated regions [28]. This process is particularly evident in hydrogels with elevated DOPA concentrations, where the increased –OH groups from DOPA likely improve water accessibility within the polymer network [29].

The selective degradation of methacrylate groups in DOPA-conjugated GelMA hydrogels suggests a tunable degradation profile that can be tailored by adjusting DOPA concentration. A controlled degradation rate is advantageous for tissue engineering applications, as it enables gradual hydrogel resorption and potential tissue ingrowth over time [30]. By modulating DOPA levels, it may be possible to create hydrogel scaffolds with precisely controlled lifespans adaptable for applications that demand specific degradation rates to support tissue maturation and remodeling. These findings suggest that DOPA-GelMA hydrogels hold significant promise for applications requiring a balance between mechanical stability and controlled biodegradation.

At a 10% GelMA concentration, NMR analysis revealed distinct differences in chemical shifts corresponding to hydroxyl groups conjugated to aromatic rings introduced by DOPA (as presented in the Supplementary Materials). Specifically, the sample with the medium DOPA concentration exhibited a net positive change in signal intensity between 4.625 ppm and 4.635 ppm, suggesting an increased presence of hydroxyl groups from dopamine incorporation. In contrast, the high DOPA concentration sample showed a net negative change in intensity within the same chemical shift range when compared to the control (no DOPA), indicating a decrease in hydroxyl group content. At first glance, these results appear contradictory. However, this phenomenon may be explained by the structural characteristics of GelMA. Due to the densely packed network of GelMA, the diffusion and reaction of DOPA with vinyl groups can be limited at lower concentrations. As the DOPA concentration increases, the probability of DOPA interacting with and reacting with the vinyl groups also rises. This reaction likely consumes both hydroxyl and vinyl functionalities, leading to the observed net decrease in intensity for the high DOPA sample. Therefore, the data support a model where the extent of DOPA incorporation is modulated by both concentration-dependent diffusion limitations and chemical reactivity within the GelMA network.

### 2.3. Rheological Properties and Potential of Injectability

**Figure 3:**
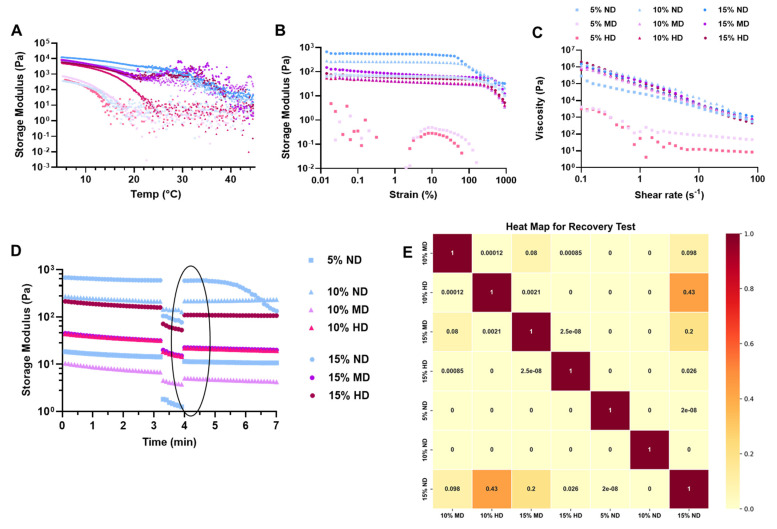
Rheological characterization of GelMA hydrogels with varying concentrations of DOPA: (**A**) temperature sweep from 5 °C to 45 °C, illustrating the influence of DOPA and hydrogel concentration on the storage (G′) and loss modulus (G″) over temperature (mean ± SD; n ≥ 3); (**B**) amplitude sweep (strain 0.01% to 100%), demonstrating the effect of DOPA conjugation on the linear viscoelastic (LVE) region of GelMA hydrogels (evolution of G′ and G″, mean ± SD; n ≥ 3); (**C**) shear-thinning behavior as a function of shear rate (0.01–100 s^−1^); and (**D**) recovery properties after injection: complete cycle including at-rest state (0.01% strain for 5 min), injection step (100% strain for 1 min), and post-injection rest state at 37 °C (strain back to 0.01% for 5 min), (the black circled area shows the recovery state of the hydrogels) (mean ± SD; n ≥ 3); (**E**) Heat map of p-values obtained from one-tailed t-tests (assuming unequal variance) comparing each pair of conditions. The analysis evaluates whether higher GelMA content improves normalized (relative to the maximum value within each sample) recovery properties.

Rheological analyses are presented in Figure 3A–E. The temperature sweep test results (Figure 3A) demonstrate a clear dependence of storage modulus on the GelMA hydrogel concentration (5%, 10%, and 15% *w*/*v*). Across all temperature ranges tested, the 15% GelMA samples exhibit the highest storage modulus values, followed by the 10% and 5% samples. This trend aligns with expectations, as higher GelMA concentrations increase the density of polymeric chains, leading to enhanced crosslinking and mechanical stiffness [15].

The inclusion of DOPA groups adversely affects the mechanical properties of the hydrogels. High DOPA-conjugated (HD) samples exhibit reduced storage modulus compared to medium (MD) and non-DOPA-conjugated (ND) samples (Figure 3A). This observation suggests that the catechol groups in DOPA, while capable of forming intermolecular interactions such as hydrogen bonding, may introduce instability or disrupt the uniformity of the crosslinked network. ND samples, lacking DOPA conjugation, demonstrate relatively higher storage modulus values, highlighting that the absence of catechol groups maintains the hydrogels’ structural integrity and mechanical stability.

The temperature-dependent behavior of the storage modulus provides insights into the hydrogel stability at both room and physiological body temperature (37 °C). The storage modulus remains relatively stable at room temperature, suggesting that the hydrogels maintain robust crosslinking networks under ambient conditions. At body temperature, a decrease in modulus is observed for most samples, indicating some degree of thermal softening or weakening of the crosslinked structure [15]. However, DOPA conjugation adversely affects the stability of hydrogels at both room temperature and physiological body temperature (37 °C). High DOPA-conjugated (HD) samples exhibit reduced storage modulus compared to medium (MD) and non-DOPA-conjugated (ND) samples, indicating that the incorporation of DOPA groups may disrupt the network structure or introduce instability under thermal conditions. This adverse effect may arise from the increased hydrophilicity or competitive interactions of catechol groups at higher temperatures, which weaken the overall crosslinking density [27,31]. Notably, the 10% HD formulation demonstrates adequate stability at both room and physiological body temperatures (Figure 3A), suggesting that at intermediate concentrations, the balance between DOPA-induced interactions and the GelMA network structure is optimized for stability.

The amplitude sweep analysis revealed significant changes in the viscoelastic properties of the hydrogels upon conjugation with DOPA groups, as shown in Figure 3B. Specifically, the conjugation led to a decrease in the storage modulus and overall stiffness of the hydrogels, which correlates with the observed reduction in the swelling ratio and enhanced biodegradation [28]. These findings suggest that DOPA conjugation may alter the hydrogel network structure, making it more susceptible to swelling and degradation. In terms of the linear viscoelastic region (LVR), all formulations, except for the 5% MD and HD variants, exhibited a well-defined LVR. The presence of DOPA groups notably extended and improved the LVR, with the yield point shifting to over 500% deformation compared to the non-DOPA (ND) samples. In contrast, the ND samples exhibited a yield point starting at around 70% deformation, indicating a significant enhancement in the deformability and stability of the hydrogel network when DOPA groups were incorporated.

All hydrogel formulations in our study exhibited shear-thinning behavior (Figure 3C), a critical property for injectable biomaterials, as it allows the material to flow more easily under stress or during application while maintaining its structural integrity at lower shear rates [32]. This finding aligns with the results reported by Ren et al. [33], who developed an ε-PLL/HA hydrogel with similar pseudoplastic characteristics. In both studies, viscosity decreased significantly with increasing shear rate, demonstrating the ability of the hydrogel networks to reorganize under flow conditions and recover post-application—a key requirement for minimally invasive delivery. All systems displayed power-law flow behavior (n < 1), characteristic of strong shear-thinning. The absence of detrimental thixotropy and the rapid viscosity drop under applied stress in our hydrogels confirm their potential for biomedical applications where ease of injection and controlled material placement are crucial.

To investigate the self-healing and recovery properties of the hydrogels after injection into a defect site, we performed a recovery test. In this test, the hydrogels were subjected to an applied force simulating the injection process, and their behavior was subsequently monitored after the removal of the applied force. The results indicated that all studied formulations (10% and 15% MD and HD) exhibited similar recovery behavior, with no significant statistical differences (Figure 3D,E). This suggests that an increase in conjugated DOPA concentration does not significantly alter the self-healing or recovery properties of the hydrogels, ensuring their potential for use in injectable applications where tissue repair and regeneration are required. The hydrogels’ ability to regain their original structure and function after deformation further supports their suitability for minimally invasive therapies [32].

### 2.4. Mechanical and Adhesive Properties

**Figure 4:**
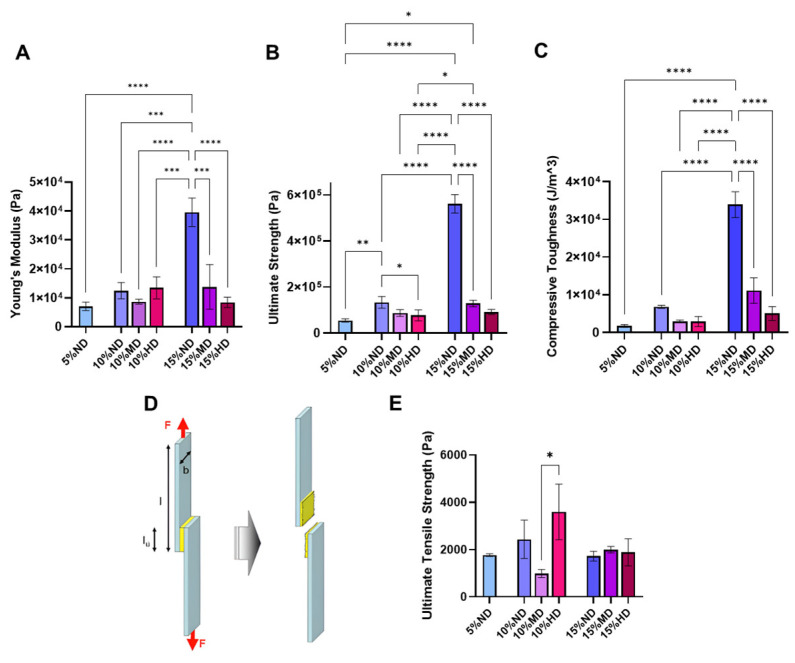
(**A**–**C**) Mechanical properties of the hydrogels under compression including Young’s modulus, ultimate strength, and compressive toughness; (**D**,**E**) adhesion properties of the hydrogels studying shear lap (n = 6, mean ± SD; * *p* < 0.05, ** *p* < 0.01, *** *p* < 0.001, and **** *p* < 0.0001; two-way ANOVA Tukey test), (an example of a stress–strain curve for the compression test of 15% MD and a force–displacement graph for the tensile test of 10% HD are presented in the Supplementary Materials).

The results presented in Figure 4A–C highlight the mechanical properties of hydrogels under compression, explicitly focusing on Young’s modulus, ultimate strength, and compressive toughness. Analyzing these results demonstrates key insights into how both hydrogel concentration and the incorporation of DOPA affect the material’s mechanical performance.

The data indicate that increasing the hydrogel concentration leads to an improvement in all three mechanical properties studied. This behavior is expected, as higher concentrations typically result in a denser network of polymer chains, providing greater deformation resistance [34]. The increase in Young’s modulus, which quantifies the material’s stiffness, suggests that a more concentrated hydrogel has a greater ability to resist elastic deformation [35]. Similarly, the ultimate strength, which measures the maximum stress the material can withstand before failure, and compressive toughness, which quantifies the material’s ability to absorb energy before fracturing, are enhanced with increased concentration [35,36]. This is consistent with the basic principle that a more concentrated polymer network generally produces stronger and more rigid materials due to higher molecular interactions and crosslinking density.

In contrast to the concentration-dependent improvements, the incorporation of DOPA (dopamine) into the hydrogel formulations resulted in a decrease in all mechanical parameters measured. DOPA is known to form non-covalent interactions, such as hydrogen bonds and coordination bonds, but it is less effective at promoting covalent crosslinking than other chemical crosslinking agents. As a result, the overall crosslinking density of the hydrogel network decreases when DOPA is added [37]. This reduced crosslinking density can explain the observed reduction in the mechanical properties. Crosslinks serve as the structural backbone of the hydrogel, providing resistance to deformation and enhancing the material’s strength and toughness [38,39]. When DOPA is incorporated, it may interfere with the formation of covalent bonds between polymer chains, resulting in a weaker and less stable network structure. Therefore, a decrease in covalent crosslinking density, due to DOPA, likely reduces the hydrogel’s stiffness, strength, and toughness.

In the 5% hydrogel concentration group, the incorporation of DOPA significantly affected the hydrogel’s ability to crosslink within 5 min. The failure of the hydrogel to crosslink properly during the testing period can be attributed to the interactions between DOPA and the polymer network. Specifically, DOPA may compete with the crosslinking agent or inhibit the formation of covalent bonds, preventing the network from fully forming in the allotted time. This results in an incomplete or poorly formed hydrogel, which cannot undergo the compression tests. This issue highlights the delicate balance between optimizing DOPA incorporation for desired functional properties (e.g., adhesion or self-healing) and maintaining the necessary mechanical integrity for testing.

In designing our bioadhesive hydrogel, we aimed to achieve an adhesion strength comparable to that of commercial fibrin glue, which is approximately 5 kPa [40]. This target is well-aligned with the typical performance of clinically relevant bioadhesives, as the majority exhibit adhesion strengths in the kilopascal range [41]. By benchmarking our material against these established values, we ensure its potential for practical biomedical applications, particularly where moderate yet effective tissue adhesion is required.

### 2.5. Cytocompatibility

**Figure 5:**
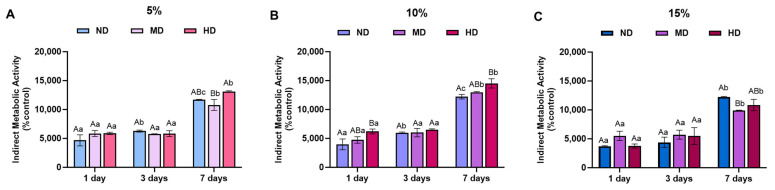
(**A**–**C**) Quantitative analysis of indirect cytotoxicity/metabolic activity (AlamarBlue^®^) in a study of the metabolic activity of Gingival Fibroblasts. The results were normalized by the positive control group (0.3% phenol red solution + culture media) for 5%, 10%, and 15% GelMA hydrogels with and without medium (MD)- and high (HD)-concentrated DOPA conjugation at Days 1, 3, and 7 (higher values indicate greater cell metabolic activity), (mean ± SD, *p* < 0.05, uppercase letters—groups within time points, lowercase letters—time points within groups; two-way ANOVA followed by Sidak’s multiple comparisons post hoc test), (based on ISO 10993-5—Biological evaluation of medical devices—Part 5: Tests for in vitro cytotoxicity) [44].

The results presented in Figure 5 focus on indirect cytotoxicity, investigating whether the release of DOPA groups or non-crosslinked methacrylated groups (functional groups on GelMA) from hydrogel formulations causes any cytotoxic effects on cells over time. The primary objective of this experiment was to evaluate the potential for cytotoxicity induced by the hydrogel formulations. Specifically, we were concerned about the release of DOPA groups or free methacrylate groups (from the GelMA polymer network) into the culture media over time. These compounds could potentially affect cell viability and metabolism if they leach from the hydrogel and enter the cell culture environment [42,43].

The absence of significant changes in cell metabolic activity at Day 3 across all hydrogel formulations suggests that, during the initial 72 h of exposure, neither DOPA nor the free methacrylated groups caused any measurable cytotoxic effects. These findings indicate that releasing these substances from the hydrogel matrix does not immediately impair cell metabolism or viability within the first three days. The non-cytotoxic behavior of the hydrogels during this period may be attributed to the relatively low release rate of the bioactive components or the hydrogels’ composition, which could minimize leaching into the media. While the degradation study was capped at 72 h to observe the initial stability and differences between formulations, the cell culture study was extended to 7 days to ensure the materials were cytocompatible over a clinically relevant timeframe. The hydrogels, particularly those with DOPA, begin to degrade within the first 72 h, but they do not completely dissolve during this period, thereby continuing to support cell viability. The 7-day cell results demonstrate that even as the material gradually degrades, it remains non-cytotoxic and can sustain cell proliferation.

By Day 7, a sharp increase in cell metabolic activity was observed across all groups. This behavior was more prominent in 5% and 10% GelMA concentrations. This is an intriguing and somewhat unexpected result, as one might anticipate a gradual decline in metabolic activity due to cytotoxicity if any toxic substances were being released. The increase in metabolic activity at this later time point could suggest several possibilities. First, the increase in metabolic activity could indicate cell proliferation or growth in response to the hydrogel components. It is possible that the hydrogels, while initially inert, begin to release small quantities of bioactive components (such as DOPA or other factors) that promote cell growth or metabolic stimulation [45,46,47]. DOPA, in particular, has been reported to exhibit bioactive properties, including promoting cell adhesion, differentiation, and proliferation, which could explain the increase in metabolic activity over time.

**Figure 6:**
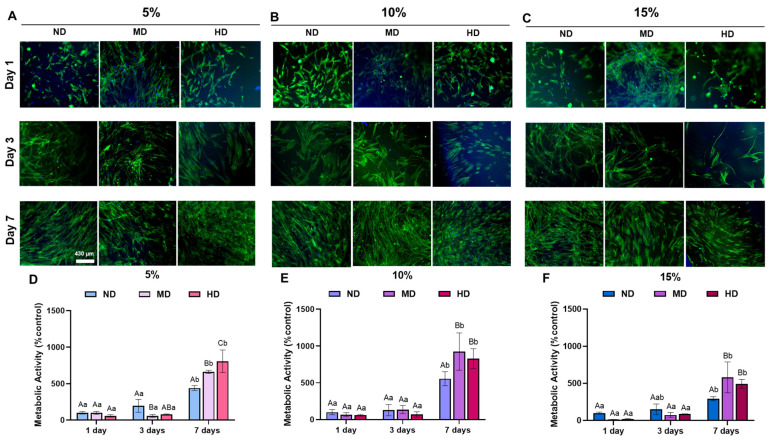
(**A**–**C**) Representative fluorescence images of Gingival Fibroblast cells seeded on the GelMA-based hydrogel at 1, 3, and 7 days, stained by F-actin (ActinGreen™ 488) and DAPI; 4× magnification, scale bar presents 430 µm (F-actin cytoskeleton staining and DAPI nuclear staining to assess cell morphology and attachment). (**D**–**F**) Quantitative analysis of direct cytotoxicity (AlamarBlue^®^) in a study of the metabolic activity of Gingival Fibroblasts. The results were normalized by the control group at Day 1 value for 5%, 10%, and 15% GelMA hydrogels with and without MD and HD conjugation at Days 1, 3, and 7 (mean ± SD, *p* < 0.05, uppercase letters—groups within time points, lowercase letters—time points within groups; two-way ANOVA followed by Sidak’s multiple comparisons post hoc test).

Another possibility is that the hydrogels begin to degrade after a specific period, releasing more substances into the culture media that can positively affect cell metabolism. For example, DOPA’s known ability to engage in self-assembly and form crosslinks could play a role in modulating cell behavior [47,48]. The findings from the direct cytocompatibility study using gingival fibroblast cells cultured on the hydrogel scaffolds (as shown in Figure 6A–F) are in alignment with the indirect cytotoxicity results in Figure 5. The AlamarBlue results show no significant variation in cell metabolic activity on Day 3, corresponding to the indirect cytotoxicity results, where no initial toxic effects were observed. This consistency suggests that the hydrogel formulations are not acutely toxic to the gingival fibroblasts in the early stages of culture. However, a notable increase in cell metabolic activity was observed on Day 7, which aligns with the earlier findings in the indirect cytotoxicity test. This increase in metabolic activity further supports the hypothesis that the hydrogels may be promoting cell growth or metabolic stimulation over time, especially considering the bioactive properties of DOPA.

The spreading and attachment of cells on the hydrogel scaffolds were analyzed on Days 1, 3, and 7 (Figure 6D–F). At Day 3, there was no significant variation in cell spreading or attachment, suggesting that the cells were able to initially interact with the hydrogel surfaces but did not yet exhibit signs of active proliferation or spreading. This could indicate that the cells adapt to the hydrogel environment during the early stages without showing substantial morphological changes. On Day 7, however, increased cell spreading and attachment were observed, suggesting that the cells had successfully integrated with the scaffold and were actively proliferating. This result is consistent with the indirect cytotoxicity results, which showed increased metabolic activity on Day 7. The improved cell spreading and attachment on the scaffolds is a positive sign of biocompatibility. It indicates that the hydrogels are non-toxic and support cell adhesion and growth over time.

In the formulation of 10% GelMA with medium and high DOPA conjugation, cell spreading and attachment were promising, with cells exhibiting better interaction with the scaffold. DOPA has been reported to enhance cell adhesion through its ability to form interactions with cell surface receptors, and its incorporation in the hydrogel likely contributes to improved spreading and attachment [48]. The bioactive effects of DOPA, particularly its capacity to engage with cell integrin and promote adhesion, likely played a key role in these positive outcomes [49].

For the 15% GelMA group, although there was an increase in cell attachment and proliferation compared to Day 3, the cell spreading appeared less favorable. The cells were observed to be more rounded, suggesting they were not fully spread out on the hydrogel surface. This rounded morphology typically indicates that the cells are not fully adhered, possibly due to inadequate interaction with the scaffold or unfavorable mechanical properties (such as stiffness or crosslinking density) [50]. This could be a sign that, although the cells could proliferate, the hydrogel’s mechanical properties (perhaps due to higher concentration or crosslinking) did not support optimal cell spreading. The rounded cells may be in a detachment-prone state, indicating reduced integration with the scaffold and a lower level of cell–matrix interaction compared to the 10% formulation.

Of note, under cell culture conditions, the hydrogels remained largely intact over 7 days, as the physiological environment is milder and degradation occurs gradually. It should be noted that the moderate degradation of the DOPA-modified hydrogels over 7 days did not negatively affect cell viability—on the contrary, metabolic activity increased, demonstrating that degradation byproducts (e.g., gelatin fragments and dopamine compounds) were biocompatible or present in non-toxic amounts.

The conjugation of dopamine to GelMA plays a critical role in modulating the hydrogel’s properties beyond what is achievable with methacrylation alone. While DOPA incorporation led to a modest reduction in mechanical stiffness and crosslinking density, it significantly enhanced adhesive capabilities due to the presence of catechol groups, which are known to promote strong interactions with tissue surfaces under wet conditions. Additionally, DOPA-modified hydrogels exhibited favorable biological responses, including increased cell adhesion and metabolic activity, particularly at intermediate GelMA concentrations. These combined effects suggest that DOPA not only compensates for reduced mechanical strength through enhanced interfacial bonding but also imparts biochemical cues that may support tissue integration, making it a valuable modification for the development of bioadhesives in regenerative applications.

## 3. Conclusions

This study demonstrates the successful conjugation of DOPA to gelatin methacryloyl (GelMA) hydrogels, resulting in enhanced adhesive and biological properties. The incorporation of DOPA led to a reduced swelling ratio and gel fraction, alongside an increased biodegradation rate, suggesting the formation of a more tightly crosslinked network. However, a decrease in overall crosslinking efficiency was also observed, likely due to competitive interactions between DOPA moieties and methacrylate groups during the crosslinking process. These findings indicate that the hydrogel system can be finely tuned to achieve controlled degradation and mechanical characteristics, making it well-suited for tissue engineering applications. Mechanical testing revealed that higher GelMA concentrations improved the hydrogel’s stiffness, strength, and toughness. In contrast, the addition of DOPA resulted in reduced mechanical performance due to a lower crosslinking density. Nonetheless, DOPA’s bioactive properties, such as enhancing cell adhesion, were evident, particularly in the 10% GelMA formulation. Cytotoxicity assays confirmed that the hydrogels were non-toxic to gingival fibroblasts during the first 3 days of culture, with increased cell metabolic activity and spreading observed by Day 7, particularly in the DOPA-conjugated formulations. These findings suggest that DOPA-modified GelMA hydrogels present a promising approach for creating biocompatible, mechanically adaptable scaffolds that support cell growth and tissue regeneration, while allowing for controlled degradation. This hydrogel is capable of rapid gelation upon blue light curing, presenting a significant advancement, as it greatly reduces patient wait time during application while minimizing the risk of damage to surrounding biological tissues.

## 4. Materials and Methods

### 4.1. Synthesis of GelMA

Gelatin type A (gel), sourced from porcine skin (gel strength: 300 g bloom) and methacrylic anhydride (MA), were obtained from Sigma-Aldrich, St. Louis, MO, USA. The synthesis of GelMA followed previously established protocols [51,52]. GelMA was synthesized by dissolving 10 g of type A gelatin in 100 mL of Dulbecco’s phosphate-buffered saline (DPBS) at 50 °C, yielding a 10% (*w*/*v*) gelatin solution. While stirring at 300 rpm, 8.0 mL of methacrylic anhydride (MA; ~8.3 g, 53.6 mmol) was added dropwise over a 5 min period. This represents a significant molar excess relative to the free amino groups (~0.8–1.0 mmol/g of gelatin), ensuring substantial methacrylation. The reaction mixture was allowed to proceed for 2 h at 50 °C. To quench unreacted methacrylic anhydride and stop the reaction, an additional 8 mL of warm DPBS was added. The resulting solution was dialyzed against deionized water (MWCO 12–14 kDa) for 7 days at 40 °C, with water changes every 12 h, to remove residual monomers and salts. The final product was lyophilized and stored at –20 °C until use.

### 4.2. Synthesis of GelMA-DOPA Conjugate

Seventy-one milliliters of MES buffer was subjected to ultrasonic degassing for 60 min. Then, for dopamine conjugation, 1 g of lyophilized GelMA was dissolved in 71 mL of MES buffer (0.1 M, pH 5.5) at 37 °C. After complete dissolution, N-(3-Dimethylaminopropyl)-N′-ethylcarbodiimide hydrochloride (EDC) (500 mg, 2.61 mmol) and N-hydroxysuccinimide (NHS) (427 mg, 3.71 mmol) were added to activate carboxyl groups on the GelMA backbone. After 20 min of stirring, dopamine hydrochloride (Sigma-Aldrich, St. Louis, MO, USA) was added as follows: for the medium DOPA conjugation (MD), 250 mg (1.32 mmol); for the high DOPA conjugation (HD), 500 mg (2.64 mmol). The following corresponds to GelMA: dopamine feed ratios of approximately 1:0.25 (*w*/*w*) for MD and 1:0.5 for HD, as illustrated in Figure 1B. The reaction was maintained at 37 °C for 24 h, protected from light. The resulting solution was dialyzed using molecular porous tubing (Spectrum Laboratories, Inc., Piscataway, NJ, USA) against acidified deionized water (pH ~3 for two hours, followed by pH ~5 for one hour) under constant stirring (1000 rpm) for a total of 3 h to remove unreacted dopamine and byproducts. The final solution was transferred to tubes, lyophilized, and stored at −20 °C [6].

To prepare the hydrogels, predetermined amounts of lyophilized GelMA-DOPA were accurately weighed and dissolved in distilled water. Once fully dissolved, lithium phenyl-2,4,6-trimethylbenzoylphosphinate (LAP) was added to the solution to serve as the photoinitiator.

### 4.3. Chemical Properties FTIR, H NMR

To evaluate the structural units of the DOPA-conjugated hydrogels, Fourier-transform infrared spectroscopy (FTIR) analysis was performed. Hydrogel samples were dried, lyophilized, and finely ground prior to analysis by Fourier-transform infrared spectroscopy (FTIR). Spectral acquisition was carried out with a Nicolet iS50 spectrometer (Thermo Fisher Scientific, Waltham, MA, USA) equipped with an attenuated total reflectance (ATR) accessory featuring a diamond crystal. The samples were placed directly onto the crystal surface and gently pressed using a low-pressure clamp to ensure consistent and effective contact. Spectra were collected by averaging 64 scans at a resolution of 2 cm^−1^, covering the spectral range from 4000 to 600 cm^−1^.

For nuclear magnetic resonance (NMR) analysis, 500 μL of deuterium oxide (D_2_O) was added to each sample to facilitate solubilization and signal acquisition. Three samples of GelMA hydrogels with a concentration of 10% (*w*/*v*) were prepared. The samples were formulated to contain varying degrees of DOPA conjugation: no DOPA (control), medium DOPA, and high DOPA content. The prepared samples were transferred into NMR tubes and analyzed using a Bruker Avance III 600 MHz NMR spectrometer (Bruker, Billerica, MA, USA) equipped with a Prodigy broadband probe. Proton NMR spectra were acquired for each sample under identical conditions to ensure comparability. The acquired NMR spectra were processed and analyzed using MestReNova software version 12. To enhance spectral resolution and minimize background signals arising from the D_2_O solvent, subtraction functions were applied between the spectra of samples with different DOPA levels. This approach allowed for the identification of spectral differences attributable to DOPA conjugation while minimizing solvent contributions.

### 4.4. Physical Properties: Swelling, Biodegradation

The swelling capacity of GelMA and GelMA-DOPA-conjugated hydrogel scaffolds was assessed following the crosslinking process by first measuring their dry weights (W_d_). The hydrogels were then incubated in 1 mL of 1×PBS (Sigma-Aldrich, St. Louis, MO, USA) at 37 °C in pre-weighed Eppendorf tubes for 24 h (n = 6/group). After this incubation period, the samples were re-weighed at 4, 24, 48, and 72 h, and the swollen weight of each sample (W_w_) was recorded. The swelling ratio was calculated using the following Equation (1) [53]:

Swelling ratio (%) = ((W_w_ − W_d_)/W_d_) × 100 (1)

After the swelling experiment, the samples were dried in a freeze dryer until they reached constant weight, after which they were re-weighed. The gel fraction was then calculated using the following Equation (2), where W_l_ represents the weight of the freeze-dried adhesive after swelling, and W_d_ denotes the initial dry weight of the adhesive [54,55]:

Gel fraction (%) = (W_l_/W_d_) × 100 (2)

GelMA and GelMA-DOPA-conjugated hydrogel scaffolds (n = 6) were prepared and immersed in PBS at 37 °C for 24 h to evaluate the degradation profile. The initial weights of the samples were recorded (W_0_). The scaffolds were then returned to PBS for specified time points (days) to measure their weights (W_t_). The degradation profile was calculated using the following Equation (3) [56]:

Degradation profile (%) = ((W_0_ − W_t_)/W_0_) × 100 (3)

### 4.5. Rheological Properties

The rheological characteristics of the hydrogels were examined using a TA Instruments rheometer with a 20 mm parallel plate (PP) geometry. Following the preparation of each hydrogel, amplitude sweep tests were conducted across a strain range of 0.01% to 100% to identify the linear viscoelastic region (LVE). The amplitude sweep test was conducted to assess the rheological properties of the hydrogels by measuring the storage modulus (G′) over a strain range of 0.01% to 500% while maintaining a constant frequency of 1 Hz. This method allowed for determining the linear viscoelastic region (LVE) and the transition point where the material behavior shifted from elastic to viscous [57]. To investigate gelation temperature, the time-dependent changes in storage modulus (G′) and loss modulus (G″) were measured within the linear viscoelastic range at a constant strain of 0.01% and a frequency of 1 Hz, while varying the temperature from 4 °C to 50 °C. The viscosity of the hydrogel solutions at 22 °C was evaluated through rotational rheometric testing. Additionally, to assess the recovery properties after injection, oscillatory tests were performed, which included a pre-injection phase (0.01% strain for 5 min at 22 °C), an injection phase (100% strain for 1 min at 22 °C), and a post-injection phase (0.01% strain for 5 min at 22 °C) [57].

### 4.6. Mechanical Properties

To investigate the effects of DOPA conjugation on the compressive properties of GelMA hydrogels, cylindrical samples with dimensions of 10 mm in diameter and 5 mm in thickness were fabricated for each experimental group. Mechanical testing was carried out at room temperature using a strain rate of 2 mm/min with an Expert 5601 testing system (ADMET, Inc., Norwood, MA, USA). The properties assessed included strain, maximum stress, and Young’s modulus.

### 4.7. Adhesion Properties

For the lap shear test, silicone sheets were utilized as substrates. A volume of 100 microliters of adhesive hydrogel was applied to one silicone sheet, after which a second sheet was placed on top of the gel to create an overlap. The assembly was then subjected to visible light curing for 5 min to ensure proper adhesion. Following the curing process, the lap shear properties of the samples were evaluated using an Expert 5601 testing system (ADMET, Inc., Norwood, MA, USA).

### 4.8. Cytocompatibility

To assess the indirect cytotoxic effects of GelMA hydrogels with and without DOPA conjugation on gingival fibroblasts, hydrogel samples (100 microliters (µL)) were prepared according to the International Organization for Standardization guidelines (ISO 10993-5 tests for cytotoxicity—in vitro methods) [44]. Gingival fibroblast cells were cultured in fibroblast medium supplemented with Epithelial Growth Factor (EGF), hydrocortisone, L-glutamine, antibiotics, antimycotics, and fetal bovine serum (FBS), and maintained in a 37 °C incubator with 5% CO_2_. The cells from passages 4 to 6 were utilized for the experiments. Six hydrogel samples per group were initially subjected to light curing for 5 min on each side to facilitate crosslinking, after which they were placed in sterile glass vials containing 5 mL of Human Fibroblast Expansion Medium supplemented with 10% FBS, L-glutamine, and 1% penicillin–streptomycin. The samples were then incubated at 37 °C, and 500 μL aliquots were collected at predetermined time points (1, 3, and 7 days) to evaluate cytotoxicity over time. To maintain a constant extraction volume, an equal volume of fresh medium was replenished in each vial after aliquot removal. The collected samples were filtered through a 0.22 μm membrane (Corning syringe filters) before being applied to the fibroblast cells for analysis [58]. Gingival fibroblasts were seeded at a density of 1 × 10^4^ cells per well on 96-well cell culture plates. After a 24 h incubation period, the medium was replaced with 100 µL of hydrogel extracts, and the cells were maintained in contact with the extracts for an additional 24 h. Cells cultured in gingival fibroblast medium without FBS served as the negative control group, while those treated with 0.3% phenol red (Sigma-Aldrich, St. Louis, MO, USA) acted as the positive control for toxicity. Following treatment, the cells were exposed to 100 µL of 10% AlamarBlue (Invitrogen, Carlsbad, CA, USA) in the FBS-free fibroblast medium for 3 h at 37 °C with 5% CO_2_. Fluorescence intensity was measured at an excitation wavelength of 560 nm and an emission wavelength of 590 nm. Cell viability was determined by converting the fluorescence readings into percentages, with the negative control at each time point designated 100%.

To evaluate the direct cytocompatibility of the hydrogel scaffolds, six samples were prepared for the assay. Each scaffold was treated with 1 mL of sterile PBS (Gibco, Waltham, MA, USA) for 10 min, followed by the addition of 1 mL of complete gingival fibroblast media, which was left for 30 min. After removing the media, 3 × 10^4^ cells were seeded onto the scaffolds. Following 1 h of initial cell adhesion, 1 mL of complete media was added to each sample. Cell viability was assessed at 1, 3, and 7 days using the AlamarBlue assay (Invitrogen, Carlsbad, CA, USA), which quantifies cell metabolism by measuring the reduction of resazurin to resofurin, resulting in a color change that correlates with mitochondrial activity [59]. After the designated culture periods, the cells were incubated with a 10% AlamarBlue solution (*v*/*v*) in FBS-free media for 3 h at 37 °C with 5% CO_2_. Fluorescence intensity was measured using a fluorometer (SpectraMax iD3, Molecular Devices LLC, San Jose, CA, USA) at excitation and emission wavelengths of 560 nm and 590 nm, respectively. Cell viability was expressed as a percentage relative to the control group (Blank GelMA) on Day 1, which was set at 100%.

### 4.9. Cell Adhesion

To evaluate cell adhesion and spreading (n ≥ 3), hydrogels were fixed at predetermined time points with 4% paraformaldehyde (Sigma-Aldrich, St. Louis, MO, USA) and then rinsed with PBS (Gibco, Waltham, MA, USA). The cells were stained for F-actin using ActinGreen 488 ReadyProbes reagent (Invitrogen, Carlsbad, CA, USA) at a dilution of 1:20 in PBS for 30 min, allowing visualization of actin filaments in green. Nuclear staining was performed with DAPI at a dilution of 1:5000 in PBS (Thermo Fisher Scientific, Waltham, MA, USA) to mark the cell nuclei. Fluorescence images of the scaffold surfaces were captured using a fluorescence microscope at 10× magnification (scale bar: 180 μm; ECHO Revolve Microscope, San Diego, CA, USA).

### 4.10. Statistical Analysis

Statistical significance among the different groups was assessed using one-way or two-way ANOVA for parametric data, conducted with Prism 9.4 software (GraphPad Software, San Diego, CA, USA). A *p*-value of less than 0.05 was considered statistically significant.

## Data Availability

The data that support the findings of this study are available from the corresponding author upon reasonable request.

## Supplementary Materials

The following supporting information can be downloaded at: https://www.mdpi.com/article/10.3390/gels11070499/s1, Figure S1: NMR results of crosslinked and non-crosslinked hydrogels with and without DOPA conjugation confirm DOPA group presence and an increase in methacrylated (MA) groups with DOPA increase (the graphs from left to right: ND, MD, HD groups with 10% (*w*/*v*) GelMA hydrogel concentration); Figure S2: (A) Stress-strain curve for compression test of 15%MD and (B) force-displacement graph for tensile test of 10%HD, presenting hydrogels’ typical behavior as a function of deformation (mean ± SD; n ≥ 3).

## Abbreviations

The following abbreviations are used in this manuscript:

DOPA	Dopamine
GelMA	Methacrylate Gelatin
