# Supplementary material for: Dopamine-Conjugated Methacrylated Gelatin Hydrogel—Physical, Mechanical, and Biological Properties
Source: Gels. 2025 Jun 26;11(7):499. doi: 10.3390/gels11070499 (PMC12294156; doi:10.3390/gels11070499)
Supplement: Supplementary file 1 [file gels-11-00499-s001.zip › gels-3637853-supplementary.pdf]

Supplementary Materials:

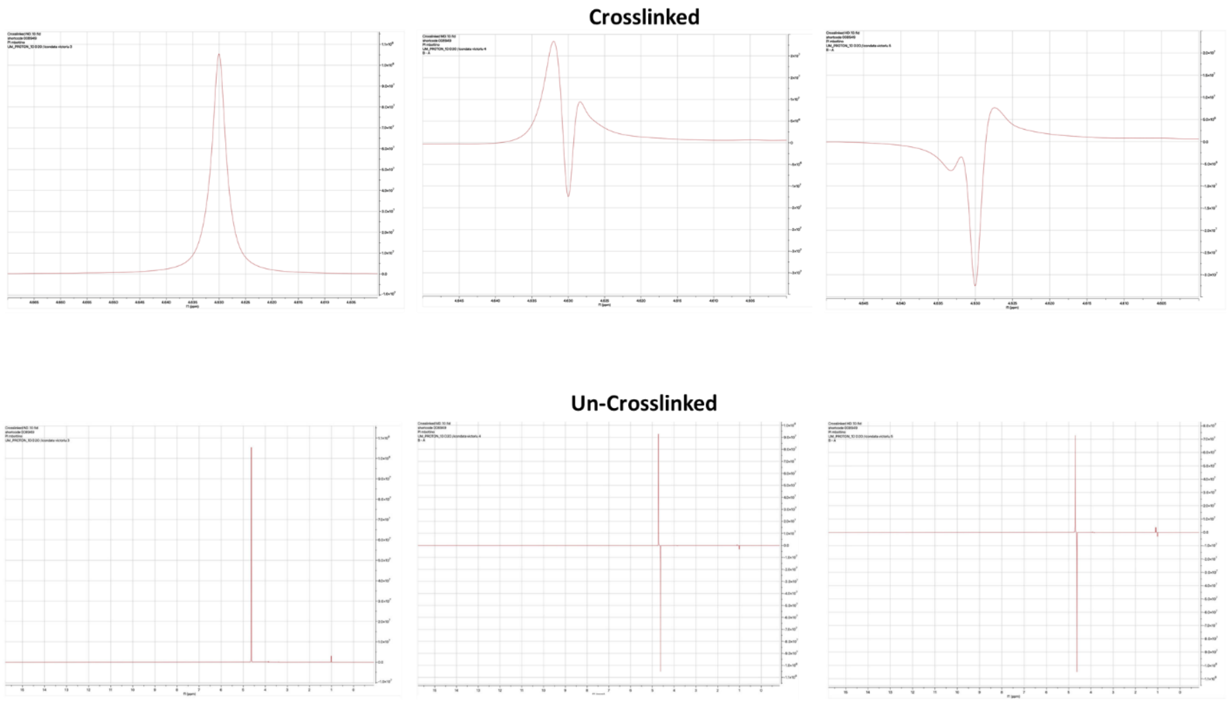

Figure S1. NMR results of crosslinked and non-crosslinked hydrogels with and without DOPA conjugation confirm DOPA group presence and an increase in methacrylated (MA) groups with DOPA increase (the graphs from left to right: ND, MD, HD groups with 10% (w/v) GelMA hydrogel concentration).

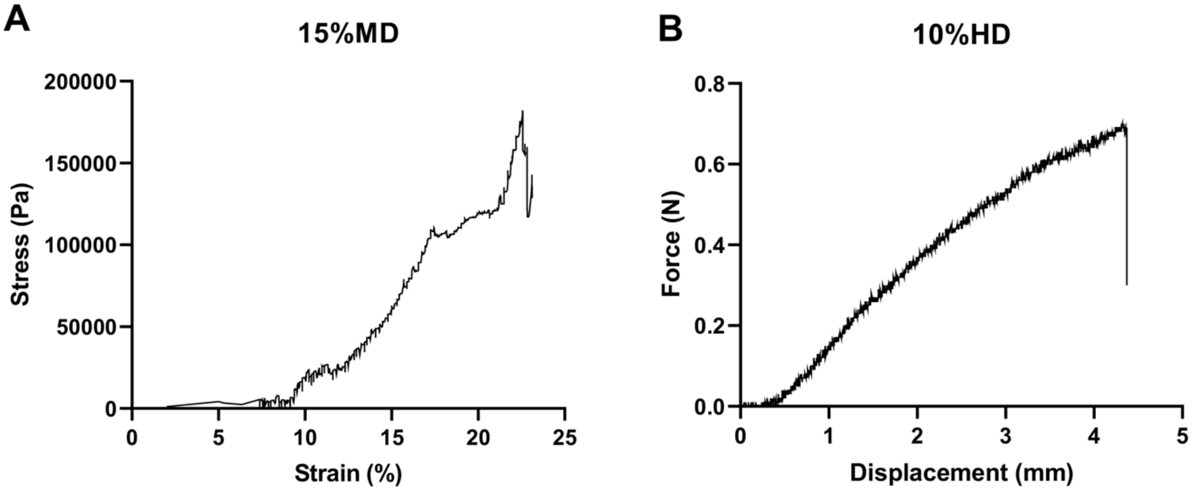

Figure SI2. A) Stress-strain curve for compression test of 15%MD and **B)** force-displacement graph for tensile test of 10%HD, presenting hydrogels' typical behavior as a function of deformation (mean ± SD; n≥3).
